# A CTB-SARS-CoV-2-ACE-2 RBD Mucosal Vaccine Protects Against Coronavirus Infection

**DOI:** 10.3390/vaccines11121865

**Published:** 2023-12-18

**Authors:** Béla Dénes, Ryan N. Fuller, Wayne Kelin, Tessa R. Levin, Jaipuneet Gil, Aaren Harewood, Márta Lőrincz, Nathan R. Wall, Anthony F. Firek, William H. R. Langridge

**Affiliations:** 1 Center for Health Disparities and Molecular Medicine, Loma Linda University School of Medicine, Mortensen Hall, Loma Linda, CA 92350, USA; denesbela@yahoo.com (B.D.); rfuller@llu.edu (R.N.F.); wkelin@llu.edu (W.K.); tessarose.levin@gmail.com (T.R.L.); jaipuneet10@gmail.com (J.G.); aarenascension@gmail.com (A.H.); nwall@llu.edu (N.R.W.); a.firek@ruhealth.org (A.F.F.); 2 Department of Microbiology and Infectious Diseases, University of Veterinary Medicine Budapest, 1143 Budapest, Hungary; lorincz.marta@univet.hu; 3 National Laboratory of Infectious Animal Diseases, Antimicrobial Resistance, Veterinary Public Health and Food Chain Safety, University of Veterinary Medicine Budapest, 1078 Budapest, Hungary; 4 Department of Basic Sciences, Oakwood University, Huntsville, AL 35896, USA; 5 Division of Biochemistry, Department of Basic Sciences, Loma Linda University School of Medicine, Loma Linda, CA 92350, USA; 6 Comparative Effectiveness and Clinical Outcomes Research Center (CECORC), Riverside University Health System Medical Center, Moreno Valley, CA 92555, USA

**Keywords:** beta coronaviruses, SARS-CoV-2, COVID-19, virus neutralization, sIgA, mucosal immunization, protein subunit vaccine, cholera toxin B subunit, angiotensin converting enzyme (ACE-2) receptor binding domain (RBD)

## Abstract

Mucosal vaccines protect against respiratory virus infection by stimulating the production of IgA antibodies that protect against virus invasion of the mucosal epithelium. In this study, a novel protein subunit mucosal vaccine was constructed for protection against infection by the beta coronavirus SARS-CoV-2. The vaccine was assembled by linking a gene encoding the SARS-CoV-2 virus S1 angiotensin converting enzyme receptor binding domain (ACE-2-RBD) downstream from a DNA fragment encoding the cholera toxin B subunit (CTB), a mucosal adjuvant known to stimulate vaccine immunogenicity. A 42 kDa vaccine fusion protein was identified in homogenates of transformed *E. coli* BL-21 cells by acrylamide gel electrophoresis and by immunoblotting against anti-CTB and anti-ACE-2-RBD primary antibodies. The chimeric CTB-SARS-CoV-2-ACE-2-RBD vaccine fusion protein was partially purified from clarified bacterial homogenates by nickel affinity column chromatography. Further vaccine purification was accomplished by polyacrylamide gel electrophoresis and electro-elution of the 42 kDa chimeric vaccine protein. Vaccine protection against SARS-CoV-2 infection was assessed by oral, nasal, and parenteral immunization of BALB/c mice with the CTB-SARS-CoV-2-ACE-2-RBD protein. Vaccine-induced SARS-CoV-2 specific antibodies were quantified in immunized mouse serum by ELISA analysis. Serum from immunized mice contained IgG and IgA antibodies that neutralized SARS-CoV-2 infection in Vero E6 cell cultures. In contrast to unimmunized mice, cytological examination of cell necrosis in lung tissues excised from immunized mice revealed no detectable cellular abnormalities. Mouse behavior following vaccine immunization remained normal throughout the duration of the experiments. Together, our data show that a CTB-adjuvant-stimulated CTB-SARS-CoV-2-ACE-2-RBD chimeric mucosal vaccine protein synthesized in bacteria can produce durable and persistent IgA antibodies in mice that neutralize the SARS-CoV-2 subvariant Omicron BA.1.1.

## 1. Introduction

Alpha coronaviruses are a family of enveloped, positive-strand RNA viruses known to cause mild respiratory and gastrointestinal (GI) tract infections [1]. While the beta coronavirus (SARS-CoV-1) and Middle East respiratory syndrome (MERS) coronaviruses cause lethal severe acute respiratory infection outbreaks [2,3,4,5,6], in late 2019, a new beta coronavirus strain, SARS-CoV-2, initiated a worldwide pandemic (COVID-19), and caused more than 1 million deaths in the U.S and 7 million deaths worldwide. Symptoms range from asymptomatic to rapid alveolar damage, respiratory failure, and death. Further studies show involvement of the lung, heart, kidney, brain, and gastrointestinal tract, suggesting that SARS-CoV-2 infection may be systemic, with long term post-acute complications leading to reduced quality of life [7]. In comparison with non-Hispanic whites, Africans, and Latinos, while being at the highest risk for death from COVID-19, are often resistant to vaccination [8,9]. In California, Latinos represent nearly two-fold more COVID-19 deaths in comparison with non-Hispanics [8].

SARS-CoV-2 contains a single 29.8 kb-plus strand RNA genome made up of eleven open reading frames that encode four structural proteins and several non-structural proteins [9]. Virus infection is mediated by the virus envelope spike glycoprotein that is composed of two subunits (S1, S2) that bind to its receptor, the host cell angiotensin-converting enzyme 2 (ACE-2), an enzyme responsible for blood pressure control and located abundantly on nasal, pulmonary alveolar, and intestinal epithelial cell membranes. The spike S1 and S2 glycoproteins were shown to be effective antigens for producing neutralizing antibody and T cell responses to SARS-CoV-2 [10]. The S1 minimal ACE-2 receptor binding domain (RBD) fragment (aa’s 333–529) was shown to be responsible for virus attachment initiating S2 mediated virus entry into the host cell [11,12,13]. In vitro binding experiments demonstrated that SARS-CoV-2-RBD binds the human ACE-2 receptor and blocks virus attachment to the cell, inhibiting virus infection [14]. The SARS-CoV-2-RBD fragment was shown to contain multiple conformation-dependent epitopes that induce strong neutralizing antibodies and CD8+ T-cell responses [14,15,16].

Injected vaccines initially generally produce high titers of pathogen specific IgG and IgA antibodies required to arrest virus infection and multiplication in infected tissues. However, overwhelming immune responses could initiate harmful excessive inflammatory responses like myocarditis, blood clots, and cytokine storm, which compromise vaccine-mediated immunity [17]. Complementation with a durable mucosal vaccine IgA response that targets epidermal cell surfaces could provide a great advantage in preventing virus infection. Mucosal vaccination stimulates plasma cell synthesis of secretory dimeric antibodies (sIgA) that undergo transcytosis through the gut mucosa into the mucus that lines the lumen of the intestine and respiratory surfaces of the lung. During and after transcytosis, these IgA antibodies intercept pathogens before they can cause infection [18,19,20,21,22,23,24]. Thus, IgA-mediated reduction in virus load could greatly assist parenteral vaccination by preventing virus infection and lowering the possibility of cytokine storm. Oral vaccine delivery to the gut mucosa stimulates mucosal sIgA production in other mucosal spaces, activating local CD8+ cytotoxic T cells and stimulating the proliferation of memory T cells in situ and at other mucosal sites, including the lung [7,25,26]. The fusion of protein adjuvants to pathogen antigens was found to increase mucosal immunity and linking the cholera toxin B subunit (CTB) mucosal adjuvant to RNA virus antigens was shown to provide well defined experimental and clinical benefits [27]. To provide an even more effective SARS-CoV-2 mucosal vaccine, fusion of the mucosal adjuvant CTB to the virus ACE-2-RBD protein antigens could amplify sIgA production, enabling increased protection against SARS-CoV-2 infection [27,28]. Because this mucosal subunit protein vaccine delivers virus epitopes directly to the mucosal immune system, it can circumvent problems caused by mRNA vaccines that hijack the cell’s machinery to synthesize and present pathogen proteins to antigen-presenting cells of the innate immune system.

One of the most serious deficiencies of parenteral vaccination is the bypassing of mucosal immunity, the primary barrier to infection at mucosal surfaces where respiratory viruses attack [29]. Because coronaviruses and other respiratory viruses like influenza and RSV enter the body through respiratory or intestinal tract epithelial cells, the initial immune response generated against the pathogen is by mucosal secretory IgA rather than humoral IgG antibodies. Thus, mucosal vaccines that traditionally stimulate a persistent IgA antibody response could help to prevent, as well as reduce or eliminate, the need for high humoral IgG antibody titers required for treatment of established virus infections. The simplicity of oral inoculation could improve vaccination compliance, particularly in economically poor countries and in African American and Latino populations reticent to accept parenteral vaccination while simultaneously being at greater risk for infection with and succumbing to the COVID-19 pandemic [8,9,29].

Our laboratory showed that a mucosal vaccine made against rotavirus enterotoxin non-structural protein #4 (NSP4) provided clinical benefits in rotavirus-infected mice and their newborn offspring. As part of the vaccine, the immuno-modulator CTB was linked to the rotavirus toxin (CTB-NSP4), and the fusion protein stably expressed in potato plants [30,31,32,33,34]. Potato tissues synthesizing the fusion protein fed to mice generated increased mucosal and fecal sIgA antibody titers in comparison with NSP4-transformed tuber tissues. Further, milk from CTB-NSP4-immunized mice protected newborn pups from rotavirus infection, and virus specific memory T cells were also identified [31]. Similar to secretory antibody prevention of rotavirus infection, and sIgA re-distribution to mucosal surfaces in the common mucosal compartment [35], oral inoculation of mice with a mucosal vaccine composed of CTB-SARS-CoV-2-ACE-2-RBD fusion proteins can provide protection to the gut and respiratory tracts from SARS-CoV-2 infection. Oral inoculation with the SARS-CoV-2 spike S1 protein provides epitopes needed for stimulation of protective B and T cell responses [36,37]. Because CTB alone was shown to stimulate sIgA production [36], its linkage to the coronavirus spike antigen may help to stimulate increased levels of sIgA. Because low levels of sIgA can prevent infection, they may also reduce inflammation and therefore provide protection against excessive inflammatory cytokine production (cytokine storm) [27]. Fusion of CTB to the SARS-CoV-2-ACE2-RBD (S1) protein may facilitate vaccine delivery across the mucosal cell membrane by binding epithelial cell GM1 ganglioside receptors. Encapsulation of the vaccine protein, as previously demonstrated in plants, could protect the vaccine from stomach acid and enzymatic degradation to ensure effective mucosal vaccine protection against virus infection [33].

For vaccine purposes, the 193-amino-acid (62.5 kDa) fragment of the SARS-CoV-2 spike (S1) angiotensin-converting enzyme (ACE2) receptor binding domain (RBD) was found to be effective in neutralizing SARS-CoV-2 with few side reactions and is therefore a prime target for subunit vaccine construction [11,14,38]. Mucosal vaccines provide a major additional advantage by inducing secretory sIgA antibodies at the mucosal surfaces of respiratory and GI tracts to prevent ingested or inhaled pathogens from binding to epithelial cells. Thus, mucosal immunity has been shown to be the first line of defense against respiratory virus infection [39,40]. Here, we demonstrate that a fusion protein generated by linking the mucosal adjuvant CTB to the coronavirus ACE-2 receptor binding domain (CTB-SARS-CoV-2-ACE-2-RBD) can produce mucosal antibody titers in mice that are protective against SARS-CoV-2 infection. Further, we establish mucosal immunization protocols that optimize IgA antibody levels effective in preventing coronavirus infection and inflammation.

## 2. Materials and Methods

### 2.1. Construction and Isolation of the CTB-SARS-CoV-2-ACE-2-RBD Vaccine Fusion Protein

**Figure 1:**
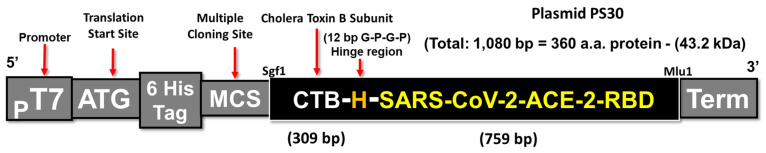
**Construction of a CTB-SARS-CoV-2 mucosal vaccine:** A 309 bp fragment of a gene encoding the cholera toxin B subunit (CTB) mucosal adjuvant was linked to the 5′ end of a DNA fragment encoding the SARS-CoV-2-ACE-2-RBD coronavirus S1 antigen. A Pro–Gly–Pro–Gly amino acid hinge region (H) was inserted between the CTB and ACE-2-RBD DNA fragments to allow molecular flexibility and to enhance ACE-2-RBD and CTB protein epitope availability for binding by antigen-presenting cells. The CTB-SARS-CoV-2-ACE-2-RBD fusion gene was inserted into the multiple cloning site (MCS) of a His-tag-based *E. coli* expression vector for nickel affinity column isolation of the vaccine fusion protein from bacterial homogenates.

**Figure 2:**
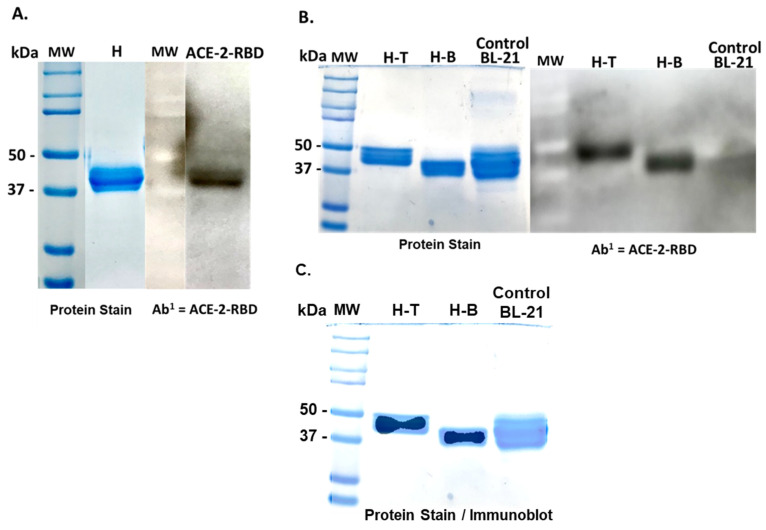
**Identification of the CTB-ACE-2-RBD fusion protein in *E. coli* BL-21 cells:** Following IPTG amplification of the vaccine protein in recombinant *E. coli* BL-21 cells, the cells were lysed, and the centrifuged homogenate proteins (H) were resuspended and separated by electrophoresis on 12% polyacrylamide gels. (Panel **A**) Lanes from left to right contain: (Lane 1) Protein molecular weight standards. (Lane 2) An approximate 42 kDa protein band excised from the homogenate gel separation and predicted to contain the CTB-SARS-CoV-2-ACE-2-RBD fusion protein was re-electrophoresed on a new gel and stained with Coomassie blue. (Lane 3) Molecular weight marker proteins negatively stained on the corresponding immunoblot. (Lane 4) Immunoblot detection of the ACE-2-RBD protein in the 42 kDa homogenate band identified by reaction with a primary human antibody (Ab^1^) made against the ACE-2-RBD protein followed by binding a mouse anti-human IgG (Ab^2^), conjugated to alkaline phosphatase. (Panel **B**) Confirmation of vaccine protein synthesis in transformed *E. coli* BL-21 cells: From left to right, (Lane 1) contains prestained protein molecular weight markers. (Lanes 2 and 3) Electro-elution of bacterial proteins in the MW range approximating 50 kDa (H-T), designated Homogenate Top band to 37 kDa (H-B) and Homogenate Bottom band. The protein bands were visualized by staining with Coomassie Blue. (Lane 4) Control untransformed BL-21 cell proteins in the same molecular weight range (50–37 kDa). (Lane 5) Negatively stained protein molecular weight markers on immunoblot of identical samples shown in the first 4 lanes. (Lanes 6–7) Immunoblot from identical proteins in lanes 2 and 3 probed with Ab^1^ antibodies specific for the human ACE-2-RBD protein. (Lane 8) Immunoblot of the control untransformed *E. coli* BL-21 cell proteins identical to lane #4 and probed with human ACE-2-RBD antibodies (Ab^1^). (Panel **C**) is identical to (Panel **B**), with the exception that the Coomassie Blue-stained homogenate lanes were overlayed with the corresponding immunoblot to demonstrate more precisely the location of the vaccine protein in homogenate proteins isolated from the transformed bacterial cells, in addition to demonstrating the absence of vaccine protein in the homogenate of untransformed bacterial cells.

An *E. coli* expression vector containing the SARS-CoV-2 subunit vaccine was assembled (Figure 1), by linking a 309 bp fragment of a gene encoding the cholera toxin B subunit (CTB) mucosal adjuvant to the 5′ end of a DNA fragment encoding the SARS-CoV-2-ACE-2-RBD coronavirus S1 antigen. A Pro–Gly–Pro–Gly amino acid hinge region was inserted between the CTB and ACE-2-RBD genes to allow molecular flexibility between the adjuvant and the spike fusion protein molecules and to enhance ACE-2-RBD and CTB protein epitope availability for identification and binding by antigen-presenting cells. The CTB-SARS-CoV-2-ACE-2-RBD fusion gene was inserted into the 5′ Sgf1 and 3′ Mlu1 cloning sites of the (MCS) region of the His-tag-based *E. coli* expression vector pEX-N-His (4.5 kb) (Blue Heron Biotech, LLC, Bothell, WA, USA) for nickel affinity column isolation of the vaccine fusion protein from bacterial homogenates. To isolate the vaccine fusion protein for immunization of mice, *E. coli* BL-21 cells (Invitrogen, Thermo Fisher Scientific, Waltham, MA, USA), containing the vaccine fusion protein plasmid CTB-SARS-CoV-2-ACE-2-RBD, were cultured over night at 37 °C in 3.0 mL of Luria Broth (LB) containing Ampicillin^100 μg/mL^ (Amp). The o/n culture (1.0 mL) was added to 250 mL of LB broth + Amp and the culture was incubated for 1–3 h to an OD_600_ = 0.4–0.5. IPTG (30 mg/250 mL culture) was then added to the culture and, four to six hours later, the bacterial culture was harvested by centrifugation at 5000 rpm for 15 min at 4 °C in a Sorvall SS-34 rotor (Thermo Fisher Scientific, Waltham, MA, USA), with the brake off. The cells were resuspended in 10 mL of lysis buffer (8.0 M Urea, 10 mM NaCl, 100 mM Tris, (pH 8.0), 1.0% SDS, 50 mM NH_4_Cl). The preparation was sonicated on ice at 5× pulses at 10 watts RMS for 10 s intervals on and 10 s off. The preparation was centrifuged in Oakridge tubes at 13,000 rpm for 30 min at 4 °C with the brake off. The supernatant was filtered through a 0.22 µm syringe filter and passed over a nickel affinity column (ProBond^TM^ Resin, Invitrogen, Carlsbad, CA, USA) to bind the histidine-containing vaccine protein. After elution of low and non-His-containing proteins with PBS, the 6-His-containing vaccine protein was eluted from the gel by competition with increasing concentrations of imidazole. The His-containing proteins were separated by gel electrophoresis of the column eluate (+/−20 ug protein per well), on 12% polyacrylamide gels with a 5% stacking gel. Independent of imidazole concentration gradients used to elute the vaccine protein, many high- or low-molecular-weight bacterial proteins remained in the column eluate. To improve vaccine protein purification, bacterial homogenates containing the vaccine protein were separated by acrylamide gel electrophoresis on 12% gels and proteins 40–50 kDa in size (MW of the vaccine protein), then were excised from the gel and electro-eluted in 48 mM Tris—39 mM glycine buffer for 4–5 h at 8–10 mA/tube in a BioRad (Hercules, CA, USA) Model 422 electroelution apparatus. The isolated proteins were again separated by electrophoresis and the separated proteins transferred to pre-wet PVDF membrane by semi-dry electrophoresis on a BioRad Trans-Blot SD Transfer Cell. Following protein transfer, primary antibodies against ACE-2-RBD, rabbit Anti-SARS-CoV-2 spike -RBD region, SAB4200874, and CTB proteins, rabbit anti-CTB antibody HPA044208–100 ul (Millipore Sigma, Burlington, MA, USA) were incubated with the blot overnight at 4 °C. The blot was washed 3× with PBS and incubated for one hour at room temp. with an anti-rabbit alkaline phosphatase secondary antibody conjugate and goat anti-rabbit IgG alkaline phosphatase conjugate A-3867 (Sigma Aldrich, St. Louis, IL, USA). The blot was washed 3 times with PBS-Tween-20 and incubated with a soluble alkaline phosphatase substrate (Invitrogen, Thermo Fisher Scientific, Waltham, MA, USA) prior to detection of light-emitting vaccine protein bands on X-ray film (X-Omat, Eastman Kodak Inc., Rochester, NY, USA),(Figure 2). 

### 2.2. Assessment of CTB-SARS-CoV-2-ACE-2-RBD Fusion Protein Purity by FPLC

**Figure 3:**
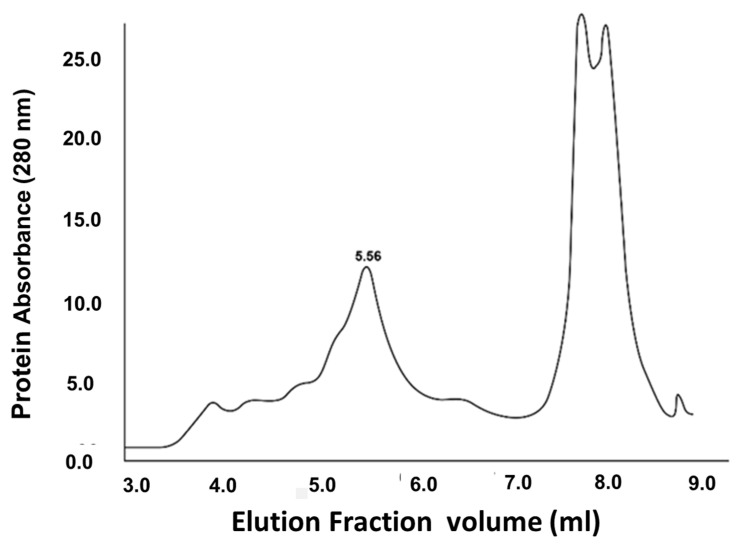
**FPLC purification of the CTB-SARS-CoV-2-ACE-RBD mucosal vaccine fusion protein:** Electro-elution of a 42–50 kDa protein band following acrylamide gel electrophoresis of transformed *E. coli* BL-21 cell homogenates may contain, in addition to the vaccine protein, bacterial proteins of a similar molecular weight. To further purify the vaccine protein, the electroeluted proteins were separated by fast protein liquid chromatography (FPLC) on two tandem Agilent SEC-5 columns (5 µm, 300 A, 4.6 × 100 mm) packed with 5 µm silica particles coated with a neutral, hydrophilic layer to aid in protein separation, connected in series and run in PBS buffer, pH 7.4, at 0.4 mL/min on a GE Healthcare FPLC system. The column eluate was monitored by UV spectrophotometry at 280 nm, and protein fractions (0.25 mL) were collected manually. The vaccine fusion protein was identified by ELISA assay in the 5.0–6.0 mL column fractions. In the assay, individual column fraction samples were bound to the wells of an Immulon 1B plate (Thermo Fisher Scientific Inc.). The vaccine protein was identified with an anti-His-Tag Mouse Monoclonal primary antibody (Thermo Fisher Scientific Inc. #37-2900) and a goat anti-mouse (H&L) peroxidase secondary antibody (Thermo Fisher Scientific Inc. #62-6520). Slower-moving molecules and molecular debris were eluted between 7.5 and 8.5 mL.

Because the vaccine protein band isolated by gel electrophoresis may contain bacterial proteins of a similar molecular weight, further purification of the vaccine protein was attempted by fast protein liquid chromatography (FPLC) separation of the vaccine fusion protein on two tandem Agilent SEC-5 Columns (5 µm, 300 A, 4.6 × 100 mm) (Agilent Technologies, La Jolla, CA, USA). The columns were packed with 5 μm silica particles coated with a neutral, hydrophilic layer to aid in protein separation. The columns were connected in series and run in PBS buffer, pH 7.4, at 0.4 mL/min on a GE Healthcare (Chicago, IL, USA) FPLC system. The column eluate was monitored via UV spectrophotometry at 280 nm, and 0.25 mL protein fractions representing the single peak were manually collected (Figure 3). The vaccine fusion protein was further identified in column fractions by an indirect ELISA in which the collected chromatographic fractions were bound to the wells of an Immulon 1B plate (Thermo Fisher Scientific). The primary antibody was an anti-His-Tag Mouse Monoclonal (Thermo Fisher Scientific #37-2900) and the secondary antibody was goat anti-mouse (H&L) peroxidase (Thermo Fisher Scientific #62-6520). A single band of approximately 42.5 kDa in size was detected (results not presented).

### 2.3. Titration of SARS-CoV-2-ACE-2-RBD Antibody Titers

In our study, we used 65 4–5-week-old BALB/c female and male mice (Charles River Laboratories, Cologne, Germany), weighing 17 g. The mice were housed in a mouse box (10 per box—to ensure statistical power) in the animal care facility of the Veterinary Diagnostic Directorate, National Food Chain Safety Office (Budapest, Hungary), at 20 ± 2 °C, 50 ± 10% humidity, and a 12 h light/12 h dark cycle with ad libitum food and drinking water. The animals were maintained in the animal care facility for at least 2 weeks prior to experiments. All animals were kept strictly in accordance with the recommendations contained in the guidelines for Care and Use of Laboratory Animals, issued by the Veterinary Diagnostic Directorate, National Food Chain Safety Office. The animal experiments were specifically approved by the ethics committee of the Veterinary Diagnostic Directorate, National Food Chain Safety Office. The animal experiment permits were issued by the Government Office of Pest County Division of Food Chain Safety, Animal Health, Plant Protection Department (Budapest, Hungary). During the experiment, the health status of the animals was monitored every day. Before the intranasal inoculation, mice were anesthetized by intraperitoneal injection of a combination of ketamine (80 mg/kg) and xylazine (5.2 mg/kg), and at the end of the experiment, before the terminal blood collection via cardiac puncture, in compliance with the National Institutes for Health Guide for the Care and Use of Laboratory Animals. All procedures with infectious materials were performed under BSL-3 conditions at the Laboratory of Virology at the Veterinary Diagnostic Directorate, National Food Chain Safety Office.

To optimize mucosal vaccine immunization, several vaccination regimes for neutralization of SARS-CoV-2 were compared. Experimental animals were divided into treatment groups of 10 mice/group (5 female and 5 male), as indicated in Table 1. The mice were immunized by oral, nasal, or parenteral (IP) injection with a 15 µg/dose of CTB-SARS-CoV-2-ACE-2-RBD fusion protein a total of 4 doses delivered at 4 wk intervals prior to serum withdrawal. In intranasal immunization 25 uL was pipetted dropwise into the alternate nares of anesthetized animals. Four weeks after the last vaccine booster dose, terminal blood samples (1–2 mL) were taken from vaccinated and control mice by cardiac puncture. RBCs were removed from the blood by centrifugation and the serum collected in Eppendorf tubes for measurement of antibody titers. A separate group of the CTB-SARS-CoV-2-ACE-2-RBD fusion protein immunized mice were boosted intraperitoneally with a total human dose (0.5 mL) of the commercially available Sputnik V (Gam-COVID-Vac (rAd5)) (Medgamal, Moscow, Russia) vaccine. Serum samples of the Gam-COVID-Vac-immunized mice were used as a control. Immunization in this separate experiment was performed intraperitoneally (0.5 mL/dose) in a prime–boost regimen: the 4-week interval between the first dose (rAd26) and the second dose (rAd5).

**Figure 4:**
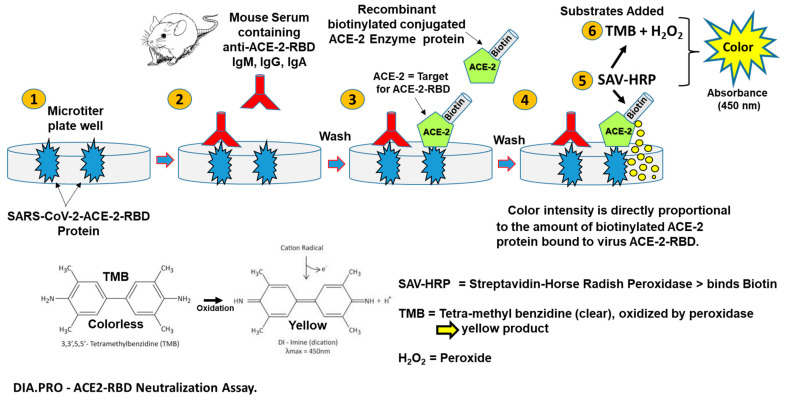
**ELISA detection of SARS-CoV-2-ACE-2-RBD-specific antibodies in immunized mouse serum:** This ELISA permits quantitative assessment of vaccine-induced antibodies that specifically neutralize the SARS-CoV-2 coronavirus. Briefly: (**1**) The wells of a 96-well microplate were coated with glycosylated ACE-2-RBD protein from the SARS-CoV-2 Omicron variant. (**2**) The ACE-2-RBD coated wells were incubated with serum dilutions from vaccine immunized mice, allowing (**3**) anti-RBD antibodies present in the serum to bind the ACE-2-RBD protein. (**4**) The plate was washed with PBS to remove excess serum, and (**5**) the remaining free ACE-2-RBD molecules were detected by incubation with recombinant ACE-2 biotinylated antigen and streptavidin-horseradish peroxidase (HRP) conjugate. (**6**) Addition of TMB + H_2_O_2_ substrates to each well initiated an oxidation reaction by HRP to convert colorless TMB to a blue color. The reaction was terminated by acidification with H_2_SO_4,_ and the now-yellow color was measured at OD 450 nm. If there are few or no antibodies in the serum to bind to the ACE-2-RBD, the color in the well will be bright yellow. However, if anti-RBD antibodies available in the serum have blocked the ability of biotinylated ACE-2 to bind the ACE-2-receptor binding domain, a color reduction or no color will be detected.

Serum samples of the Gam-COVID-Vac-immunized mice were used as a control. Immunization in this separate experiment was performed intraperitoneally (0.5 mL/dose) in a prime–boost regimen: the 4-week interval between the first dose (rAd26) and the second dose (rAd5). A sensitive ELISA assay (ACE-2-RBD Neutralization Assay, DIA.PRO Diagnostic Bioprobes Srl, Milan, Italy) was used according to the manufacturer’s instructions to measure SARS-CoV-2-ACE-2-RBD virus-receptor-specific antibody titers in the serum of immunized mice (Figure 4). Briefly, the wells of a 96-well microplate were coated with the SARS-CoV-2 recombinant glycosylated ACE-2-RBD protein. The ACE-2-RBD-coated wells were incubated with serial dilutions of serum from vaccine-immunized mice, allowing anti-RBD specific antibodies present in the serum to bind and inactivate the ACE-2-RBD protein. After washing the wells with PBS to remove the serum, any ACE-2-RBD molecules that remained free were detected by addition of recombinant ACE-2 biotinylated antigen and streptavidin–horseradish peroxidase (HRP) conjugate. The addition of TMB + H_2_O_2_ substrates to each well enabled HRP oxidation of colorless TMB to a green–yellow color, with an absorbance maximum at OD_450_ nm. If few or no antibodies in the serum bind to RBD, the color in the well will be bright yellow. Conversely, if serum anti-RBD specific antibodies have blocked biotinylated ACE-2 binding to the receptor binding domain, a reduced color or no color is detected. Using this ELISA, serum from each mouse was tested for the presence of antibodies specific for binding to and inactivating the SARS-CoV-2- ACE-2-RBD receptor protein.

### 2.4. ELISA Determination of Vaccine-Induced ACE-2-RBD-Specific IgA

To make a quantitative assessment of specific antibody levels in animals and their ability to neutralize SARS-CoV-2 infection in vivo, anti-virus IgA antibody levels were quantified in the sera of CTB-SARS-CoV-2-ACE-2-RBD mucosal-vaccine-immunized mice. The wells of a 96-well MaxiSorp™ ELISA plate (Thermo Fisher Scientific Inc., Waltham, MA, USA) were coated with recombinant SARS-CoV-2-ACE-2-RBD protein (Sigma-Aldrich, St. Louis IL, SAE 1000-50UG-PW) and incubated with serum from immunized mice overnight. Serum IgA levels were detected by addition of a goat anti-mouse IgA alpha chain (HRP) conjugate (Abcam, Cambridge, UK). The enzyme activity was visualized by HRP oxidation of tetramethylbenzidine (TMB). The optical density (OD_450_) of samples was measured spectrophotometrically.

### 2.5. Mucosal Vaccine Neutralization of SARS-CoV-2 Infection In Vitro by a Virus Neutralization (VN) Assay

To determine mucosal vaccine neutralization of SARS-CoV-2 in vivo, Vero E6 African green monkey kidney cells in log phase growth were transferred into the wells of a 96-well tissue culture plate (Thermo Fisher Scientific Inc., Waltham, MA, USA) at 10,000 cells/well, the day before coronavirus infection. Serum samples from immunized and unimmunized mice were mixed with an equal volume of 100 × TCID50% of the SARS-CoV-2 (Omicron variant (B.1.1.529). Two-fold serial dilutions of the serum/virus mixture were added to microplate wells containing the Vero E6 cells at a growth stage of 80–90% confluency. The assay was performed in triplicate. The Vero E6 cell line was obtained from the American Type Culture Collection (ATCC) and was cultured in Minimum Essential Medium Eagle culture medium (MEM) (Sigma-Aldrich, St. Louis, IL, USA) with 10% fetal bovine serum (FBS) and 2 mM L-glutamine and antibiotic-antimycotic solution (all obtained from Sigma-Aldrich). The infected cells were incubated at 37 °C in a 5% CO_2_ incubator. Virus neutralization titers were determined at 4 dpi (days post infection), as observed in an inverted microscope. Serum neutralizing titers were read as the highest dilution of the serum where the cytopathic effect remained above 50%.

### 2.6. Detection of Anti-ACE-2-RBD Antibodies in Lung Alveolar Tissues

Paraffin-embedded tissue sections of vaccinated and unvaccinated mouse lung alveolar tissues were incubated for 16 h at 37 °C with PEG-8000 precipitated SARS-CoV-2 Hungarian isolate (SARS-CoV-2 human /HUN/CMC1/2020, GenBank OQ302121.1, 1:1000 dilution). The slides were washed 5 min in PBS to remove unbound virus. The sections were incubated with SARS-CoV-2 IgA- and IgG-positive human sera (1:10 dilution), which was collected from an unvaccinated individual during the 1st wave of the pandemic, for 1 h at 37 °C. The slides were washed as before, and the sections incubated with anti-human IgG-HRP conjugate (Abcam, Cambridge, UK, 1:1000 dil.) for 1 h at room temp. After washing for 5 min to remove excess Ab^2^, the sections were stained with chromogen stain (EnVision Flex HRP Magenta substrate, Dako Omnis (Glostrup, Denmark) Code Nr: GV925) and inspected for the presence of virus specific antibodies by light microscopy.

### 2.7. Statistical Analysis

Figures with statistics were built in Rstudio (build 351) using ggplot2 (3.4.2) (Wickham H (2016). ggplot2: Elegant Graphics for Data Analysis. Springer-Verlag New York. ISBN 978-3-319-24277-4, https://ggplot2.tidyverse.org). Nonparametric Wilcoxon tests were used through the stat_compare_means function in ggplot2 to determine *p*-value significance between compared groups. The number of animals represented in each figure and statistical analysis are highlighted within each treatment group. *p*-values are represented as * = *p*-value < 0.05, ** = *p*-value < 0.01, *** = *p*-value < 0.001, **** = *p*-value < 0.0001, and NS = *p*-value > 0.05.

## 3. Results

### 3.1. Construction of a CTB-SARS-CoV-2 Fusion Protein Subunit Mucosal Vaccine

To stimulate a persistent protective mucosal antibody immune response against the SARS-CoV-2 coronavirus and its variants, a protein subunit mucosal vaccine containing the mucosal adjuvant CTB linked to the SARS-CoV-2 ACE-2- receptor binding domain (RBD) was assembled as described in Figure 1.

### 3.2. Isolation and Purification of a CTB-SARS-CoV-2-ACE-2-RBD Fusion Protein Mucosal Vaccine

The presence of both CTB and ACE-2-RBD proteins were detected at the same molecular weight on immunoblots of transformed *E. coli* BL-21 cells (Figure 2A,B), indicating the vaccine protein is approximately 42 kDa in size, corresponding to the molecular weight calculated for the fusion protein. While the vaccine fusion protein was partially purified by (Ni++) metal affinity column chromatography, washing the Ni column with an increasing gradient of imidazole did not significantly increase the level of purity of the vaccine protein eluted from the column and resulted in a continuous loss of vaccine protein with increasing imidazole gradient elution steps. In contrast, electro-elution of the 42 kDa protein from preparative 12% acrylamide gels resulted in a large increase in the amount of vaccine protein isolated and allowed purification directly from clarified bacterial homogenates (+/−200 µg/250 mL *E. coli* BL-21 cells), without significant contamination, with high- or low-molecular-weight bacterial proteins. Isolation of the vaccine protein from the few remaining contaminating proteins of similar size was attempted by FPLC (Figure 3)**.** The vaccine fusion protein identified by ELISA assay in column fractions from 5.0–6.0 mL. Slower-moving unrelated molecules and molecular debris eluted from the column between 7.5 and 8.5 mL.

However, the column protein isolation procedure substantially increased the extracted protein volume and revealed a small shoulder to the right of the main vaccine peak, suggesting the presence of a protein of potential bacterial origin or a possible minor variation in the structure of the vaccine protein (Figure 2B). The absence of detectable CTB or ACE-2-RBD protein in immunoblots of untransformed bacterial cells confirms the absence of bacterial or other non-specific proteins that could generate a signal at the same molecular weight as the vaccine protein. No further purification of the vaccine protein was attempted.

### 3.3. Optimization of CTB-SARS-CoV-2-ACE-2-RBD Mucosal Vaccine Immunization

**Figure 5:**
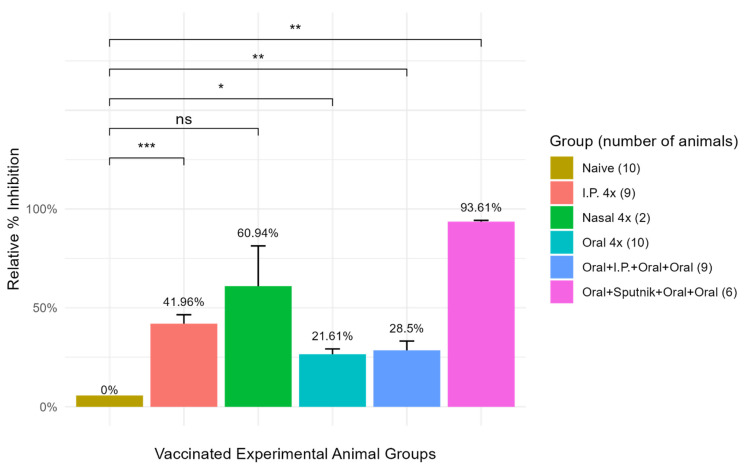
**Optimization of SARS-CoV-2-ACE-2-RBD specific antibody levels.** A sensitive virus neutralization ELISA assay (DIA.PRO ACE-2-RBD) was used to compare several immunization protocols for optimum production of SARS-CoV-2-ACE-2-RBD antibodies in coronavirus-vaccinated mice. Mouse anti-virus antibody inhibition of biotinylated ACE-2 protein binding to the virus ACE-2 receptor binding domain (OD_450_ reduction) is presented on the Y axis. All vaccination protocols produced significant ACE-2-RBD-specific antibody levels in comparison with the unimmunized control. 4x-refers to delivery of four separate immunizations to the mice at intervals of 3–4 weeks between immunizations. Statistical analysis of the data from each experimental group was compared with the control group by a nonparametric Wilcoxon test where probability values ranged from * = *p*-value < 0.05, ** = *p*-value < 0.01, *** = *p*-value < 0.001, ns = *p*-value > 0.05. The error bars represent SEM.

A sensitive ELISA assay (ACE-2-RBD Neutralization Assay, DIA.PRO, Milan, Italy) was employed to determine the optimum mucosal immunization strategy for generating SARS-CoV-2-ACE-2-RBD-specific antibody production in mice, as illustrated in the following cartoon (Figure 4). Serum isolated from immunized mice in each vaccination group was reacted with recombinant SARS-CoV-2-ACE-2-RBD protein bound to ELISA plate wells to permit quantification of antibodies that specifically bind to the virus ACE-2-RBD receptor protein (Figure 5). The resulting data show that all mucosal vaccination protocols produced significant levels of ACE-2-RBD-specific antibodies in comparison with naïve unvaccinated mice. Priming mice with the oral vaccine and boosting with a Sputnik V (Gam-COVID-Vac) vaccine injection (pink bar) generated the greatest level of anti-ACE-2-RBD-specific antibodies, providing a 94% inhibition of virus receptor binding in comparison with the unvaccinated control. Intranasal immunization (green bar) generated a 61% inhibition, providing the highest level of antibodies produced by mucosal vaccination alone. Vaccine injection into the intraperitoneal cavity (IP) (orange bar) generated a 42% inhibition in virus binding. Oral immunization plus an oral or IP boost (light blue bar and dark blue bar respectively) provided only a 22–29% inhibition, the smallest difference between vaccinated and unvaccinated animals. Comparison of the data generated from each experimental mouse group with the naïve control group by a nonparametric Student’s *t*-test gave probability values that ranged from * = *p*-value < 0.05, ** = *p*-value < 0.01, *** = *p*-value < 0.001; corresponding confidence intervals (CI) ranged from * *p* = 95% CI, ** *p* = 99% CI, *** *p* = 99.9% CI. The error bars represent standard error of the mean (SEM). Together, these data suggest that mucosal vaccine protein synthesized in bacteria stimulates virus receptor specific antibody production in mice that can neutralize the coronavirus through attachment to the virus S1 ACE-2-RBD receptor binding domain. In comparison with serum antibody levels measured in mice parenterally immunized with Sputnik V, coronavirus-specific antibody levels generated in mice following oral immunization with CTB-SARS-CoV-2-ACE-2-RBD mucosal vaccine were about 3–4 times lower than those in the Sputnik-vaccinated mice.

### 3.4. Mucosal Vaccination Generates Virus Specific IgA Antibodies

**Figure 6:**
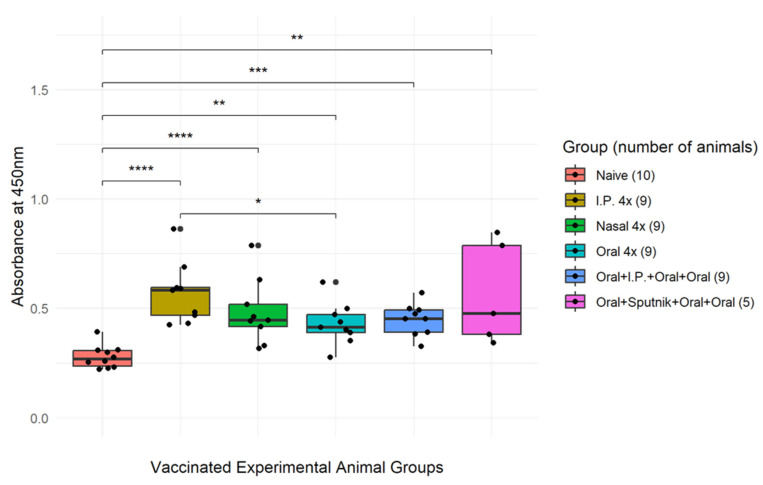
**Mucosal vaccination generates virus specific IgA antibodies.** Several vaccination regimes were examined by an indirect ELISA assay for generating optimum titers of SARS-CoV-2-RBD-specific IgA antibodies. Microtiter plate wells coated with the ACE-2 receptor protein were incubated with undiluted serum from mice immunized by oral, nasal, and IP injection routes. 4x refers to delivery of four separate immunizations to the animals at an interval of 3–4 weeks between each immunization. Virus-receptor-specific IgA antibody levels were detected by incubation of the wells with goat anti-mouse IgA alpha chain conjugated to horseradish peroxidase (HRP). Peroxidase activity was detected by addition of TMB substrate and H_2_O_2_ to each well and the color reaction measured at OD_450_ nm. (Increases in OD_450_ = greater amounts of IgA). In box plot format, the IgA titration data for each treatment group was divided into quartiles (25% of the population). The boxes represent the second and third quartiles. The horizontal bars represent the median values for the group, with 25% of the group above and 25% below the median separating the second and third quartiles. The whiskers represent the first and last quartiles. The black dots are individual animal IgA OD_450_ absorbance values. Analysis of the data by Wilcoxon test comparing individual vaccination methods to the naive unvaccinated control indicated that IP injection (brown box) was the most effective immunization protocol for generating maximum IgA levels. Comparison among vaccinated groups by Wilcoxon test indicated one significant difference in IgA antibody levels between the IP injected and Oral immunized groups. The asterisk is used to denote the level of statistical significance associated with a *p*-value. (* = *p*-value < 0.05, ** = *p*-value < 0.01, *** = *p*-value < 0.001, **** = *p*-value < 0.0001).

Several vaccination regimes were compared by the SARS-CoV-2 ELISA assay for generating optimum titers of SARS-CoV-2-RBD-specific IgA antibodies (Figure 6). Microtiter plate wells coated with the ACE-2 receptor protein were incubated with undiluted serum from mice immunized by oral, intra-nasal, and IP injection routes. Virus-receptor-specific IgA antibody levels were detected by incubation of the wells with goat anti-mouse IgA alpha chain conjugated to horseradish peroxidase (HRP). Peroxidase activity was detected by addition of TMB substrate and H_2_O_2_ to each well and the color reaction measured at OD_450_ nm. (Increases in OD_450_ = greater amounts of IgA). In box plot format, the IgA titration data for each treatment group was divided into quartiles (25% of the population). The horizontal bars represent the median values for the group with 25% of the group above and 25% below the median. The black dots are individual animal IgA OD450 absorbance values. Analysis of the data by Wilcoxon test comparing individual vaccination methods to the naive unvaccinated control indicated that IP injection (brown box) was the most effective immunization protocol for generating maximum IgA levels. Comparison among vaccinated groups by Wilcoxon tests indicated one significant difference in IgA antibody levels between the IP-injected and Oral immunized groups. (** = *p*-value < 0.01, *** = *p*-value < 0.001, **** = *p*-value < 0.0001).—[*p* = < 0.05 = 95% confidence interval (CI), *p* = < 0.1–99% CI, *p* = < 0.001–99.9% CI,—*p* = < 0.0001 = 99.99% CI].

### 3.5. Virus Neutralization Is Enhanced by Combined Mucosal and Parenteral Immunization

**Figure 7:**
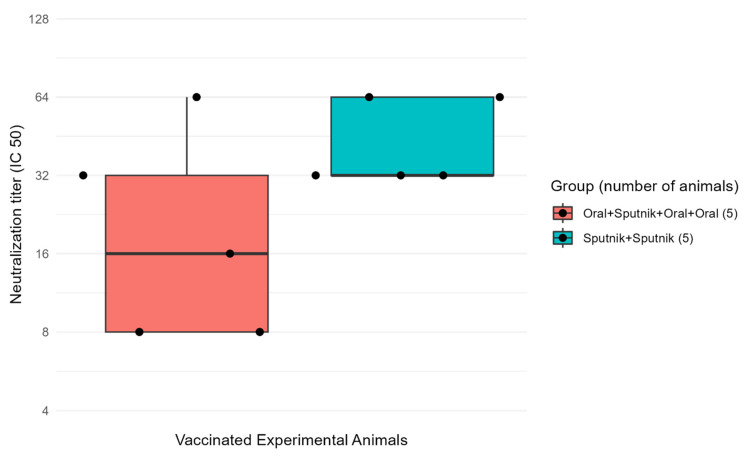
**The combination of mucosal and parenteral immunization enhances virus neutralization.** Coronavirus neutralizing antibody titers were evaluated microscopically by a virus neutralization assay (VN) in Vero E6 Green Monkey kidney cells. In this box plot graph, the Y axis represents serial dilutions of mouse serum starting from a ¼ dilution of the serum. The X axis displays individual mice in each group that generated virus-neutralizing serum titers (black dots). In the **Blue box**, the mice were primed and boosted at 4 weeks by IP injection with the Sputnik V DNA vaccine. In the **Red box**, the mice were orally primed with 15 µg mucosal vaccine and boosted once by IP injection of the Sputnik 5 vaccine followed by 2× oral gavage with 15 µg mucosal vaccine protein at 4 wk intervals. In this graph, the samples are divided into quartiles. The boxes represent the second and third quartiles, with the black horizontal line in each box, the sample median, separating the second and third quartiles. The whiskers represent the first and last quartiles. The Oral + Sputnik V + Oral + Oral group (Red box) showed a minimum effective titer at 1:8 and a maximum at 1:64. The Sputnik V immunization group (Blue box), showed a minimum effective titer at 1:32 and a maximum at 1:64.

Virus-neutralizing antibody titers were determined in Vero E6 green monkey cells from one experimental group of mice primed and boosted once by IP injection with the Sputnik V DNA vaccine (Figure 7, blue box). A second group of mice were primed by oral inoculation with 15 µg of mucosal vaccine protein followed by IP injection with the Sputnik V DNA vaccine and two additional oral boosters of 15 µg of vaccine protein delivered at 4 wk intervals (Figure 7, red box). The Y axis indicates mouse serum neutralization dilutions. The X axis indicates individual mice in each experimental immunization group that generated measurable virus neutralizing serum titers (black dots). The boxes represent the second and third quartiles. The black horizontal line in each box represents the sample median. The whiskers on the top and bottom of the boxes represent the first and last (4th) quartiles. All mice from the Sputnik V + Sputnik V group and Oral + Sputnik V + Oral + Oral groups expressed effective viral neutralization titers. The Sputnik V prime + Sputnik V booster IP immunization group (blue box) showed effective neutralization titers as low as 1:32 dilution and as high as 1:64 serum dilutions. The Oral + Sputnik V + Oral + Oral group (red box) showed a minimum effective titer at 1:8 and a maximum at 1:64. The group treated orally with CTB-SARS-CoV-2-ACE-2-RBD vaccine combined with injected Sputnik V had more variable effective titers in comparison with the group with Sputnik V IP priming with a Sputnik V IP booster. The neutralizing titers were read as the highest dilution where the cytopathic effect is still above 50% (IC-50).

### 3.6. Mucosal Vaccine Neutralization of SARS-CoV-2

**Figure 8:**
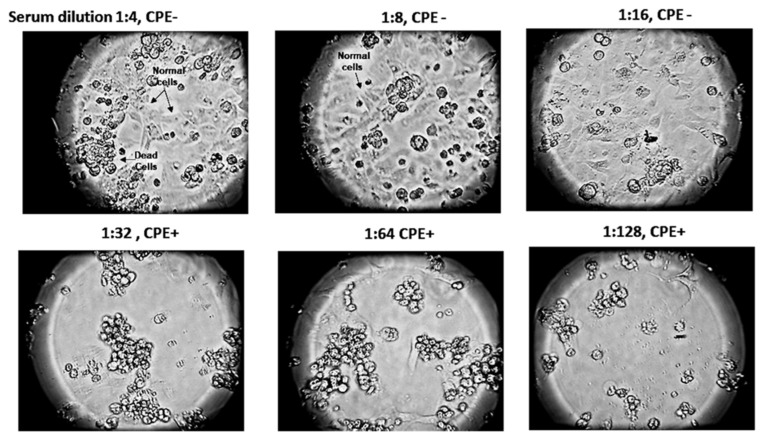
**Oral vaccine neutralization of the SARS-CoV-2 Omicron variant B.1.1.529.** Serial dilutions of serum harvested from oral-vaccine-immunized mice were employed to neutralize a constant amount of SARS-CoV-2 Omicron virus variant. In this assay, vaccine-immunized mouse serum + corona virus samples were added to Vero E6 African green monkey kidney cells in tissue culture plates. After several days’ incubation of cell cultures infected with the virus + serum dilution mixtures, the level of virus neutralization was determined by light microscopy, where the titer was read as the highest serum dilution where the detected cytopathic effect (CPE) of cell necrosis was above 50%. As vaccine titers were decreased, there was observed a concomitant decrease in the number of Vero cells (1:32 serum dilution). Further, as vaccine titers were reduced, increased clusters of apoptotic cells were detected (1:32–1:64 serum dilutions), allowing the virus to infect more cells. Disappearance of the background lawn of normal attached Vero cells was seen to dramatically increase as virus-infected cells became necrotic, clumped together and lysed (1:32–1:128 serum dilutions). Microscope magnification = 100×.

Serial dilutions of serum from orally immunized mice were used to neutralize the SARS-CoV-2 Omicron variant B.1.1.529 in Vero E6 African green monkey cells (Figure 8). Virus neutralization titers were determined by phase contrast microscopy, read as the highest serum dilution where the cytopathic effect (cell necrosis and lysis) exceeds 50%. The highest serum antibody titer found to neutralize the coronavirus was between 1:32 and 1:16. In comparison with IgG titers, IgA titers detected are frequently significantly lower. The relatively low IgA titer detected may be an immune response to the need for relatively low IgA titers in the gut lumen for effective inhibition of the relatively low virus load frequently present during virus infection of the intestinal mucosae.

### 3.7. Detection of SARS-CoV-2 Specific Antibodies in Vaccinated Mouse Lung Alveolar Tissues

**Figure 9:**
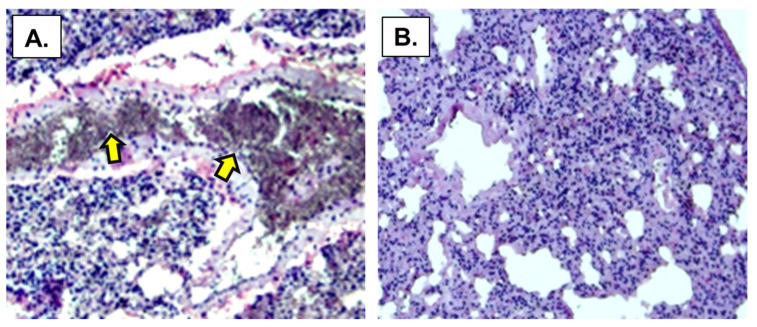
**Immunohistochemical detection of SARS-CoV-2 specific antibodies in vaccinated mouse alveolar lung tissues.** Representative paraffin-embedded lung alveolar tissue sections from CTB-SARS-CoV-2-ACE-2-RBD protein vaccinated BALB/c mice Panel (**A**) and unvaccinated mice Panel (**B**). Slides containing several mouse lung alveolar tissue sections (10µm) were incubated for 1 h with the Hungarian SARS-CoV-2 virus isolate SARS-CoV-2/human/HUN/CMC1/2020, GenBank OQ302121.1 wt., at a virus dilution of 1:1000. After virus incubation, the slides were washed extensively in DI water to remove unbound virus from the sections. After washing, the sections were incubated for 1 h with human anti-SARS-CoV-2 antibodies (1:10 dilution). Following antibody incubation, the sections were washed several times to remove unbound antibodies. Finally, the sections were incubated with anti-human IgG-HRP conjugate (Abcam, 1:1000 dilution) for 1 h at room temp. The sections were then incubated with chromogen stain (EnVision Flex HRP magenta substrate, Dako Omnis Code Nr: GV925). Representative tissue sections were inspected for virus-specific antibody bound to residual virus by light microscopy. In Panel (**A**), section areas with dark red–purple stain (indicated by arrows), indicate the presence of coronavirus-specific antibodies binding residual coronavirus particles in virus-inoculated lung alveolar tissue sections from immunized mice. In Panel (**B**), the absence of antibody staining indicates no coronavirus-specific antibodies are present in virus-inoculated lung alveolar tissue sections from unimmunized mice. Microscope magnification = 400×.

Paraffin embedded tissue sections from nasal immunized (Figure 9A) and unimmunized mouse lung alveolar tissues (Figure 9B) were incubated with SARS-CoV-2, Hungarian isolate (SARS-CoV-2 /human/ HUN/CMC1/2020. GenBank OQ302121.1) and then with human anti-SARS-CoV-2 antibodies. The slides were counterstained with an anti-human IgG-HRP conjugate and finally with a chromogen substrate. Inspection of the tissue sections for the presence of the virus by light microscopy showed areas of dark purple staining (arrows), indicating that the virus was retained in the tissue section by virus-specific antibodies generated following nasal immunization with the mucosal vaccine. No SARS-CoV-2 staining was detected in the unimmunized mouse lung alveolar tissues. The retention of exogenously added coronavirus (SARS-CoV-2) in the immunized mouse and the lack of virus retention of the added SARS-CoV-2 demonstrates that mice immunized with the CTB-SARS-CoV-2-ACE-2-RBD vaccine generate lung alveolar-tissue-specific anti-SARS-CoV-2 antibodies.

## 4. Conclusions 

The presence of CTB-SARS-CoV-2-ACE-2-RBD vaccine fusion protein in transformed *E. coli* BL-21 cells (Figure 2A) is confirmed by the lack of an ACE-2-RBD protein band in untransformed bacterial cells (Figure 2B). The presence of a predominant major peak at the approximately correct location during FPLC purification of the vaccine protein suggests that electro-elution of the vaccine protein from bacterial homogenates generates a relatively pure protein product Figure 3. However, the presence of a small shoulder on the vaccine protein band during FPLC separation suggests the possibility of vaccine molecules with an altered structural conformation, a different state of glycosylation, or potentially a foreign protein of bacterial origin. Because there was no evidence of obvious adverse health effects detected in the organs or behavior of the mice immunized with our vaccine fusion protein, these observations suggest that the vaccine may be safe for applications in humans. However, no clinical evidence was provided in this study to further substantiate this claim. A TMB substrate color reduction of more than 50% in orally immunized mice in comparison with unimmunized mice (Figure 5) demonstrates significant serum-specific antibody binding to the virus ACE-2-RBD antigen. However, virus-specific antibodies in the serum of mice parenterally immunized with the Sputnik V DNA vaccine were 3–4 times greater. This result might be expected, as mucosal immunization often generates significantly lower levels of immunity in comparison with parenteral vaccination. The different routes of mucosal immunization showed that intranasal immunization generated the highest levels of virus-specific antibodies. Because only several mice were immunized, the standard error was large Figure 5. Technical issues related to intranasal administration of the vaccine, including degradation of vaccine protein in the nasal cavity, could be responsible for the large variability observed in the mouse responses to nasal immunization. For intra-nasal immunization, the vaccine protein was delivered in PBS rather than in bicarbonate buffer used for oral immunization. The requirement for neutralization of acid in the gut could alter mucosal vaccine stability, resulting in a reduction in vaccine efficacy. The impact of neutralization of stomach acid and vaccine protein digestion are areas requiring additional experimentation. However, the robust immune response generated by nasal administration of the vaccine in comparison with oral vaccine immunization is an observation confirmed in many immunization studies.

Interestingly, all modes of mucosal immunization appeared to be statistically equivalent in producing virus-specific antibodies, except for parenteral vaccine delivery, which produced significantly higher levels of ACE-2-RBD-specific antibodies in comparison with oral immunization (Figure 6). Vaccine efficacy may be affected by glycosylation of the antigen protein, an important determinant. Bacterial synthesized vaccine proteins may remain un-glycosylated or possess a pattern of “O” or “N” glycosylation that is different from glycosylation patterns generated in eukaryotic cells. Thus, differences in the post-translational modification (glycosylation pattern) on the virus antigen protein synthesized in *E. coli* may produce antibodies that have diminished specificity, affinity to or reactivity with the virus ACE-2-RBD-receptor antigen. To assess the role of vaccine protein glycosylation in vaccine efficacy, producing the vaccine protein in an alternative eukaryotic cell system, e.g., plants, will be pursued. Because vaccine specificity, structure, function, stability, and immunization efficacy all depend, to a significant extent, on the pattern of sugars post-translationally bound to the vaccine protein, we predict increased levels of antiviral antibodies and that their affinity for the virus antigen will be produced in mice immunized with mucosal vaccine produced in eukaryotic organisms. where vaccine protein glycosylation patterns more closely resemble eukaryotic glycosylation patterns.

Oral vaccine priming followed by a single parenteral Sputnik V DNA vaccine boost demonstrated that ACE-2-RBD specific antibody titers can be significantly increased. The similarity between virus neutralization titers of mice immunized by parenteral plus mucosal vaccine immunization and priming and boosting with the DNA vaccine alone (Figure 7) suggests mucosal immunization enhances and can replace booster doses of the more costly DNA vaccine to provide equivalent levels of protection against beta coronavirus infection at a reduced cost. These data may lead to a viable clinical strategy in future vaccine campaigns. Thus, a single parenteral DNA vaccine dose with repeated oral boosting could deliver durable high levels of protective (IgA) antibodies, providing continuous immune protection against virus infection or reinfection. Mice orally primed and boosted with the CTB-SARS-CoV-2-ACE-2-RBD mucosal vaccine protected Vero E6 African green monkey kidney cells from coronavirus infection (Figure 8). Demonstrating the flexibility of mucosal vaccine immunization, oral inoculation of the intestinal mucosa provided immune protection of the lung against infection by SARS-CoV-2 and other respiratory viruses [41,42]. 

Because mucosal vaccines remain stable at ambient temperature and do not require trained personnel for vaccine delivery, substitution of parenteral vaccine boosters with oral, intranasal, or even parenteral delivery of the CTB-SARS-CoV-2-ACE-2-RBD vaccine could significantly improve current problems of vaccine dissemination. Although parenteral delivery of the CTB-SARS-CoV-2-ACE-2-RBD vaccine generally produced significantly higher levels of anti-ACE-2-RBD-specific antibodies than oral immunization (Figure 7), because the amount of virus passing through the mucosal epithelial cell barrier is often low in comparison with virus replication in the body’s cells, fewer mucosal IgA antibodies may be required to protect against infection by intercepting virus passing through mucosal epidermal cells into the circulation. Thus, oral immunization may be as effective or even more effective than parenteral vaccination in halting coronavirus infection when one compares the amount of circulating IgG antibodies required to halt a virus infection. Thus, an “ounce of prevention” of virus penetration into the circulation may be more than equivalent to the “pound of cure” delivered by peritoneal immunization.

Unexpectedly, efforts to identify IgG titers against the virus receptor binding domain stimulated by mucosal immunization with the vaccine were too variable for reliable detection. In addition, not all animals in each vaccination group provided measurable antibody titers. What may be responsible for this observation remains unknown. However, no negative behavior modifications or cytological detection of tissue or organ damage (cell necrosis) was observed in any of the mice in experimental groups following oral, nasal, or parenteral vaccination regimes employing the CTB-SARS-CoV-2-ACE-2-RBD vaccine. Because vaccine specificity, structure, function, stability, and immunization efficacy depend significantly on the pattern of sugars post-translationally bound to the vaccine protein, we predict increased levels of antiviral antibodies and that their affinity for the virus antigen will be produced in mice immunized with mucosal vaccine produced in eukaryotic organisms like edible plants, where vaccine protein glycosylation patterns are of eukaryotic origin.

In summary, we demonstrate that a protein subunit vaccine containing a mucosal adjuvant physically linked to a dominant SARS-CoV-2 coronavirus surface receptor antigen produced a specific antibody response against the virus by various mucosal routes of administration. These experimental results indicate that the mucosal immune system responds to this novel vaccine protein by generating virus-specific IgA antibodies. When synthesized in bacteria, the vaccine protein generates virus specific antibody levels in mice that protect against infection by the SARS-CoV-2 coronavirus, but validation in whole animal coronavirus infection studies remains to be completed. Further, we show that oral immunization with the CTB-SARS-CoV-2-ACE-2-RBD vaccine in combination with parenteral virus DNA vaccination can provide more effective and economic protection against SARS-CoV-2 infection than can be generated by parenteral or mucosal immunization alone.

## Figures and Tables

**Table 1:** Mouse Treatment Groups.

Group	Delivery	Vaccine Treatment (Total of 4 Doses at 4-Week Intervals)	Dose
1 (n = 10)	Oral	(4×) Bicarbonate buffer (negative control)	0.5 mL
2 (n = 10)	Oral	(4×) 15 μg of CTB-SARS-CoV-2–ACE2-RBD in Bicarbonate Buffer	0.5 mL
3 (n = 10)	Parenteral (i.p.)	(4×) 15 μg of CTB-SARS-CoV-2–ACE2-RBD in PBS	0.3 mL
4 (n = 10)	Oral + Parenteral + Oral + Oral	(Oral 3×) 15 μg of CTB-SARS-CoV-2–ACE2-RBD in Bicarbonate Buffer (i.p. 1×) 15 μg of CTB-SARS-CoV-2–ACE2-RBD in PBS	0.5 mL 0.3 mL
5 (n = 10)	Nasal	(4×) 15 μg of CTB-SARS-CoV-2–ACE2-RBD in Bicarbonate Buffer	0.05 mL
6 (n = 10)	Oral + Parenteral + Oral + Oral	(Oral 3×) 15 μg of CTB-SARS-CoV-2–ACE2-RBD in Bicarbonate Buffer (i.p. 1×) Sputnik V (full human dose)	0.5 mL 0.5 mL

## Data Availability

Further data available upon request.
